# Assessing the Relationship between Systemic Immune-Inflammation Index and Metabolic Syndrome in Children with Obesity

**DOI:** 10.3390/ijms24098414

**Published:** 2023-05-08

**Authors:** Delia-Maria Nicoară, Andrei-Ioan Munteanu, Alexandra-Cristina Scutca, Niculina Mang, Iulius Juganaru, Giorgiana-Flavia Brad, Otilia Mărginean

**Affiliations:** 1 Department of Pediatrics, University of Medicine and Pharmacy “Victor Babes”, 300040 Timisoara, Romania; nicoara.delia@umft.ro (D.-M.N.); scutca.alexandra@umft.ro (A.-C.S.); nina.mang@umft.ro (N.M.); juganaru.iulius@umft.ro (I.J.); giorgiana.brad@gmail.com (G.-F.B.); marginean.otilia@umft.ro (O.M.); 2 Department of Pediatrics I, Children’s Emergency Hospital “Louis Turcanu”, 300011 Timisoara, Romania; 3 Department XI Pediatrics, Discipline I Pediatrics, Disturbances of Growth and Development in Children–BELIVE, 300011 Timisoara, Romania

**Keywords:** obesity, metabolic syndrome, children, inflammation, biomarker

## Abstract

Childhood obesity represents a worldwide concern as many countries have reported an increase in its incidence, with possible cardiovascular long-term implications. The mechanism that links cardiovascular disease to obesity is related to low-grade inflammation. We designed this study to investigate the diagnostic utility of inflammatory indices (NLR, neutrophil-to-lymphocyte ratio; PLR, platelet-to-lymphocyte ratio; SII, systemic immune-inflammation index; SIRI, systemic inflammation response index) in obese children with metabolic syndrome (MetS) and their relationship with cardiometabolic risk biomarkers, such as the Homeostasis Model Assessment of Insulin Resistance (HOMA-IR), triglyceride-to-high-density lipoprotein cholesterol (TG:HDL-C), and non-high-density lipoprotein cholesterol (non-HDL-C). A total of 191 obese children from one large Romanian reference center was included in the study. Patients were classified in two groups according to the presence (MetS group) or absence (non-MetS group) of metabolic syndrome. According to our results, the SII index proved to have diagnostic value in distinguishing MetS patients among children with obesity (AUC = 0.843, a sensitivity of 0.83, and a specificity of 0.63). Furthermore, the SII was positively associated with cardiometabolic risk biomarkers (HOMA-IR, *p* < 0.001; TG:HDL-C, *p* = 0.002; non-HDL-C, *p* = 0.021), highlighting its possible role as an additional measure of cardiometabolic instability in obese children.

## 1. Introduction

Childhood obesity is regarded as a worldwide concern as many countries have reported an increase in its incidence greater than the one seen in adults [1]. According to the World Health Organization (WHO), in 2016, 41 million children under 5 years of age were overweight or obese and over 340 million children and adolescents aged 5–19 were overweight or obese [2]. Furthermore, studies focused on adults reported an increase in disorders linked to obesity, such as insulin resistance, type 2 diabetes mellitus, non-alcoholic fatty liver disease, cardiovascular disease, stroke, cancer, and autoimmune disorders [3,4,5]. In Romania, one in four children over the age of six was found to be either overweight or obese [6]. A growing body of studies has linked childhood abdominal obesity to heightened cardiometabolic risk and pointed out early structural changes, such as left ventricular hypertrophy and increased carotid intima-media thickness [7,8,9,10,11,12,13].

In an attempt to assess this cardiovascular risk and guide the management of obese children, pediatricians have increasingly diagnosed metabolic syndrome (MetS) [14,15]. Over 40 definitions of pediatric MetS have been proposed by different organizations and authors stemming from adult MetS criteria [16,17]. Each one similarly includes central obesity, dyslipidemia, hypertension, and altered glucose metabolism, but they differ with respect to the cut-off points and weighting of various components [14,18,19,20,21]. Importantly, MetS is linked to several possible associations, such as cardiovascular disease, hepatic steatosis, diabetes mellitus, obstructive sleep apnea, and polycystic ovarian syndrome [22]. Although the exact pathogenic pathways behind MetS remain incompletely elucidated, the interaction between obesity, low-grade inflammation, and insulin resistance seems to play a key role in its development [23,24,25,26].

In the last decade, different indices have proven their value in estimating clinical and subclinical peripheral inflammation, including the neutrophil-to-lymphocyte ratio (NLR) [27], platelet-to-lymphocyte ratio (PLR) [28], systemic immune-inflammation index (SII) [29,30,31,32], and systemic inflammation response index (SIRI) [33]. However, most of the studies using these indices have focused on adult populations. 

We therefore designed the present study to assess the relationship between complete blood count (CBC)-derived inflammatory biomarkers and the presence of MetS in obese children.

## 2. Results

### 2.1. Patient Characteristics

According to a retrospective chart review, we identified 329 children with obesity. A final amount of 138 patients was excluded because of insufficient data, active infections, or medical conditions known to alter hematological parameters. Thus, the study population consisted of 191 patients, 80 females (41.9%), and 111 males (58.1%). The median age was 13 (interquartile range (IQR): 11–15) years old. 

Patients were divided into two categories: those that fulfilled the metabolic syndrome criteria (MetS group, *n* = 66) and those that did not (non-MetS, *n* = 125). The prevalence of MetS was 34.5%. Both the MetS and non-MetS groups were similar in terms of age and gender. As expected, both the anthropometric parameters and median blood pressure values were significantly elevated among the children with MetS (*p* < 0.001). Table 1 summarizes the comparison of the clinical and laboratory features between the two study groups. 

There were significantly higher WBC, neutrophil, and thrombocyte values in the MetS group (*p* < 0.001), whereas lymphocyte and monocyte counts were similar to those without MetS (*p* = 0.789 and *p* = 0.103, respectively). 

In terms of lipid and glucose profiles, both groups had similar cholesterol (*p* = 0.148) and LDL-C (*p* = 0.431) levels, while glycemia (*p* = 0.034), insulinemia (*p* < 0.001), and triglycerides (*p* < 0.001) were more elevated and HDL-C (*p* < 0.001) was significantly lower in the MetS group. Moreover, the three cardiometabolic indices that were assessed displayed significant increases in the MetS group (<0.001).

**Figure 1:**
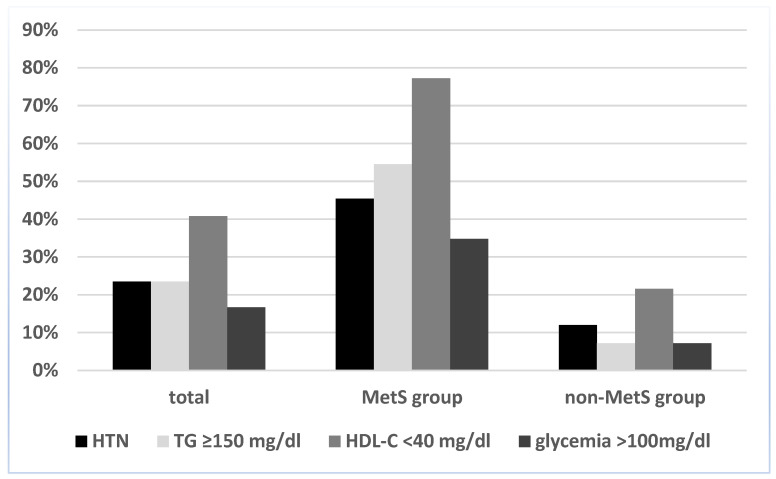
Prevalence of individual MetS components in the study lot, according to the International Diabetes Federation (IDF) definition. Abbreviations: HTN, hypertension; HDL-C, high-density lipoprotein cholesterol; TG, triglyceride.

Regarding the prevalence of each MetS component among the entire obesity group, the most prevalent one was low HDL-C (40.8%), followed by hypertension and elevated TG levels (both with a 23.5% prevalence). The least frequent metabolic syndrome component was hyperglycemia, present in only 16.7% of children, as can be seen in Figure 1.

### 2.2. CBC-Derived Inflammatory Parameters

Regarding blood-derived inflammatory indices, we observed that each parameter displayed a significant increase in MetS patients, as revealed by the independent sample *t*-test in Table 2.

**Figure 2:**
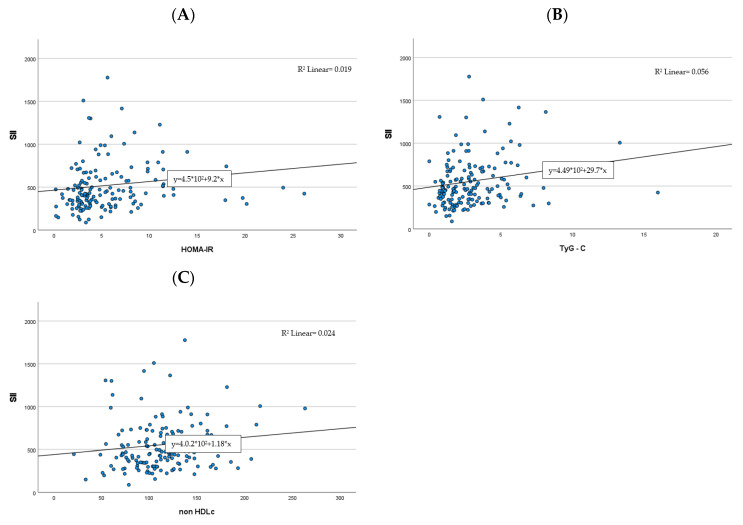
Correlations between the SII index and cardiometabolic risk factors ((**A**)—HOMA-IR, (**B**)—TG:HDL-C, (**C**)—non-HDL-C). 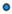, case number; 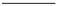, regression.

Moreover, out of the four CBC-derived indices, SII was the only one to positively correlate with all cardiometabolic markers used in the study (see Table 3, Figure 2, and the Supplementary Materials Table S1). 

### 2.3. Association between SII Index and MetS

Binary logistic regression analysis was used to further assess the relationship between the SII and presence of metabolic syndrome in our study groups (Table 4), with MetS as the dependent variable and the SII, HOMA-IR, TG:HDL-C ratio, and non-HDL-C as the independent variables. In this analysis, the SII and TG:HDL-C were the only indices significantly related to MetS (*p* < 0.001).

The association between the SII and individual components of metabolic syndrome was investigated by means of multiple regression analysis. As depicted in Table 5, waist circumference was the only individual component significantly associated with the SII.

### 2.4. ROC Curves of Inflammation and Cardiometabolic Markers

**Figure 3:**
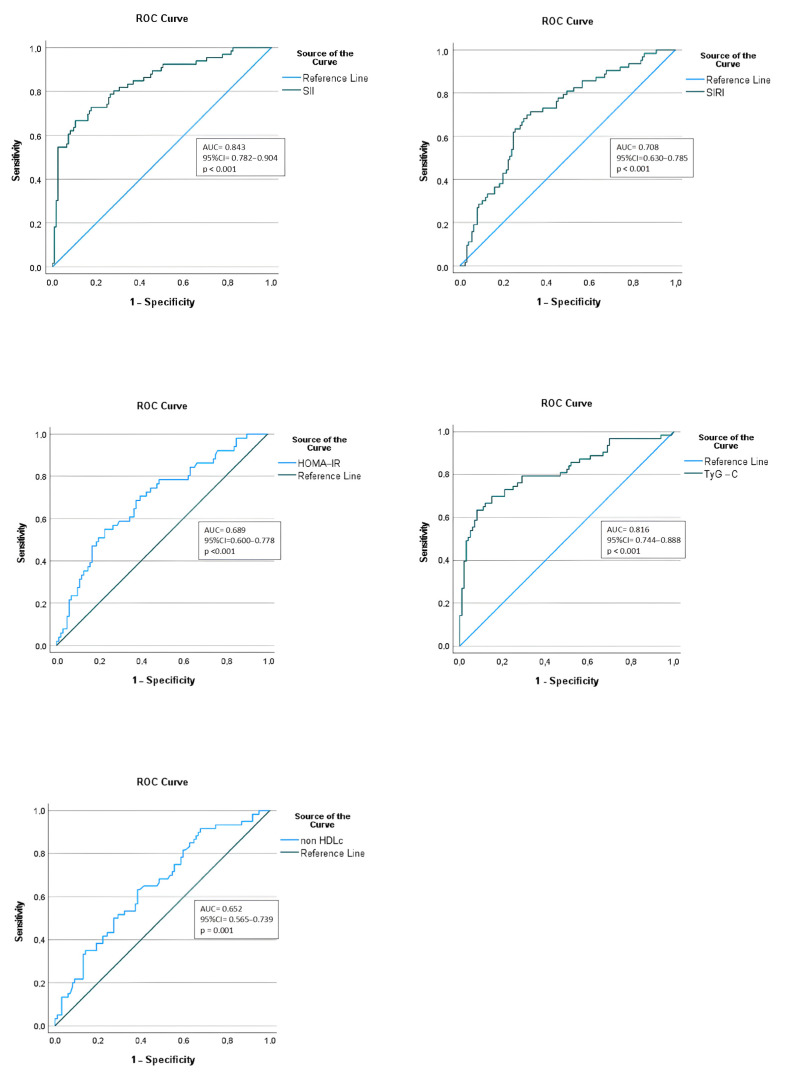
Diagnostic performance assessment of inflammatory and cardiometabolic indices in predicting MetS in obese children.

We further assessed the diagnostic value of the SII in correctly identifying MetS patients, by performing an ROC curve analysis. We compared it with the predictive accuracy of the HOMA-IR, TG:HDL-C index, and non-HDL-C. Based on the data summarized in Table 6 and Figure 3, the SII was found to have the highest discriminative capacity for metabolic syndrome, with an optimal SII cut-off point of 426 × 10^3^ and an area under the curve (AUC), sensitivity, and specificity of 0.843, 0.83 and 0.63, respectively. According to the ROC curve analysis, a similar discriminative capacity was observed for TG:HDL-C, with an optimum cut-off point of 1.786 and a sensitivity of 0.81, but with lower specificity (0.50).

## 3. Discussion

The most important finding of this study was that the SII index proved to have diagnostic value in distinguishing MetS patients among children with obesity and was associated with cardiometabolic risk biomarkers, highlighting its role as an additional measure of metabolic instability in obese children.

Since the mention of cardiovascular risk factor clustering in children in 1999 [34], there has been a growing interest among pediatricians in the premature recognition of various components of MetS in overweight and obese children in order to prevent future health complications [24]. Contrary to adult populations, there is still debate regarding the most accurate MetS definition [15,35], which could explain the discrepancies regarding MetS prevalence among children in different studies [36]. According to a systematic review by Friend et al. based on 85 studies on pediatric populations, the median prevalence of metabolic syndrome among obese children ranged between 10 and 66% [37]. In our study lot, 34.5% of children had MetS. One possible explanation for the rather low prevalence in our study group could be the chosen criteria for MetS definition. There are several diagnostic criteria used for detecting MetS proposed by different organizations and authors, such as the IDF, WHO, National Cholesterol Education Program’s Adult Treatment Panel II (NCEP-ATP III), Cook et al., Ferranti et al., Viner et al., and Weiss et al. [14,19,20,38]. Overall, studies using Ferranti et al. criteria usually registered the highest prevalence (4.0–26.4%), while those using IDF criteria provided the lowest number of children diagnosed with MetS (0.3–9.5%) [39].

Regarding the prevalence of individual MetS components among the study lot, our results were similar to those from the literature [40,41,42,43], with dyslipidemia being the most frequently encountered anomaly in 40% of cases and hyperglycemia the least frequent, in 16.7%. As expected, the prevalence of each MetS component was significantly higher in children that met the criteria for metabolic syndrome. Surprisingly, although we used the IDF criteria which propose adult cut-off points for blood pressure and thus result in a lower prevalence of hypertension compared to other definitions [44,45,46,47], almost one third of patients had elevated blood pressure. This could be, in part, attributed to the presence of a cardiology unit among the department from which our patients originated and thus a greater addressability of children with cardiovascular complaints. 

Previous studies have adressed the cardiometabolic risk that concerns children and adolescents with obesity, especially those with a greater MetS load [48,49]. According to the Princeton Lipid Research Clinics Follow-up Study, pediatric MetS was a significant predictor for cardiovascular disease and type 2 diabetes mellitus in adulthood [50]. A study by Chinial and coworkers described significant right atrial dilation, increased left heart chambers, and left ventricular hypertrophy in adolescents with MetS [51]. Given the plasticity of the pediatric cardiovascular system that proves able to reverse damage in the presence of timely interventions, identifying and treating children with cardiovascular risk factors is of great importance [52].

In the last decade, it has gradually been proven that systemic inflammation initiates and aggravates the pathological process of chronic diseases [53,54,55,56,57]. There is an increasing trend towards evaluating the potential value of hematological pro-inflammatory markers in the diagnosis and prognosis of various chronic diseases [58,59,60,61] given the link between inflammation and changes in peripheral blood cells. Aside from the classical inflammatory biomarkers, such as C-reactive protein, white blood cells, and neutrophils, the most studied complete blood cell indices include NLR and PLR [62,63,64,65,66]. Such cost-effective markers are readily available from routine blood tests and provide significant information regarding systemic inflammation status [67]. 

Compared to the available indices that use only one or two blood cell subtypes, the SII integrates all three major immune cell lineages [68] to better reflect the complexity of the inflammatory response [69,70,71,72,73,74,75]. Furthermore, it is relatively stable despite the alteration in physiological conditions [71]. Since the first report in 2014 by Hu et al. describing it as a useful prognostic index in hepatocellular carcinoma [29], the SII has been widely used in oncology [71,75,76] and in different inflammatory conditions [77]. These have included cardiovascular diseases such as coronary artery disease [78,79], hypertension [80], and acute ischemic stroke [81] and chronic HF [82], autoimmune diseases, diabetes, and obesity [83,84].

However, there is insufficient evidence regarding the SII in the context of low-grade inflammation associated with pediatric MetS, which promotes cardiovascular disease. This low-grade inflammation is part of the linking mechanism between obesity and metabolic syndrome [85]. Bastard et al. concluded in an article published in 2006 that low-grade chronic inflammation present in obese patients leads to insulin resistance through numerous pathways [86]. Previous studies also reported that the presence of pediatric MetS may predict future cardiovascular risk, due to the association between metabolic syndrome and inflammation [1,50,87]. 

Therefore, in the present study, we assessed the diagnostic value of the SII in reflecting the presence of metabolic syndrome among children with obesity and its association with well-known cardiovascular risk biomarkers. According to our study, the subjects from the MetS group had higher levels of inflammation status, as depicted by elevated WBC, neutrophil, thrombocyte, NLR, PLR, SII, and SIRI (*p* < 0.001) values. This is in agreement with adult studies, such as the large population study conducted by Liu et al. in 2019 [88], and the one reported by Buyukkaya et al. which found a significant correlation between MetS criteria and inflammation depicted by NLR [89,90]. Similar results were provided by Akboga et al. regarding PLR, stating that elevated index values were significantly associated with the presence and severity of MetS [91]. As opposed to the data from adult studies, those regarding the relationship between the SII and childhood MetS are very limited.

We found one study by Öztürk et al. [92] that investigated the NLR, PLR, and SII in children with metabolic syndrome and their relationship with macular damage in MetS children. They reported that SII levels were more elevated in obese children, especially those with metabolic syndrome (*p* = 0.021). Additionally, regarding the cardiometabolic risk profile, multiple studies have emphasized the value of composite biomarkers such as the triglyceride-to-high-density lipoprotein ratio and non-HDLc in predicting atherogenic lipid profiles and insulin resistance [93,94,95,96,97,98]. In our study lot, we noticed that SII was significantly correlated with the TG/HDLc index and non-HDLc. Children from our MetS group had a median TG/HDLc value of 4.13, consistent with a higher risk for cardiovascular diseases, as implied by previous studies that associated ratio values above 3 in children with a higher risk of CVD [99,100]. This finding is in agreement with previous studies in adult populations, which state that systemic inflammation indices such as the SII are associated with a higher risk for cardiovascular diseases [101,102,103,104]. The results were further confirmed by using binomial logistic regression that depicted the SII as an independent risk factor for metabolic syndrome (*p* < 0.001). Additionally, since the SII had the largest area under the curve in the ROC analysis, it can be argued that the SII is a better index for discriminating obese children with and without MetS than other CBC-derived indices, similar to the HOMA-IR, TG/HDL ratio, and non-HDL-C. 

Altogether, the SII index tended to better reflect the presence of MetS, outperforming other indices such as the NLR and other individual components of the SII index, and was positively correlated with cardiometabolic risk biomarkers. It is important for practitioners to identify patients at risk of cardiovascular disease in a timely manner because of the plasticity of the cardiovascular system seen in children, which renders it reversible to damage if treated promptly and effectively [51].

However, there are some potential limitations to interpreting our results that need to be addressed. First, the retrospective cross-sectional nature of this study rendered our observations susceptible to inherent bias and causality could not be established, allowing for only an interpretation of associations. Secondly, there emerged a relatively low number of cases and the absence of a normal weight control group from the retrospective collection of data. In addition, validated but not routinely used inflammatory markers, including cytokines, were not studied, as they are not routinely tested in our hospital. Therefore, prospective studies with a larger cohort size are required to provide additional information.

## 4. Materials and Methods

### 4.1. Study Design and Patient Selection

This retrospective cross-sectional study was conducted in one of the largest Romanian reference pediatric centers. Medical records of 329 consecutive patients diagnosed with obesity in the Pediatric Emergency Hospital “Louis Turcanu” from Timisoara, Romania, between 1 January 2015 and 28 February 2023, were analyzed in accordance with the Declaration of Helsinki (1975, revised in 2013). Our study protocol was approved by the Local Ethics Committee; informed consent was waived, due to the retrospective nature of the study. Inclusion criteria were as follows: (1) age range between 10 and 18 years and (2) diagnosis of obesity. Exclusion criteria included: active infections, medical conditions known to alter hematological parameters, and patients with incomplete data regarding the anthropometric measures or laboratory data. Obesity was diagnosed according to the WHO guidelines (WHO Reference 2007 for older children and adolescents) as having a BMI-for-age greater than 2 standard deviations above the WHO growth reference median [105]. Metabolic syndrome was defined using the IDF criteria, as central obesity (waist circumference ≥ 90th percentile) plus at least two additional criteria: (1) TG ≥ 150 mg/dL, (2) HDL-C < 40 mg/dL, (3) systolic blood pressure ≥ 130 mmHg or diastolic blood pressure ≥ 85 mmHg, and (4) fasting plasma glucose (FG) ≥ 100 mg/dL or previously diagnosed type 2 diabetes, in children between 10 and 16 years [17]. The patients were divided into two groups, according to the presence (MetS group) or absence (non-MetS group) of metabolic syndrome.

### 4.2. Clinical and Laboratory Assessments

The following clinical data were collected: demographic characteristics (age, gender) and anthropometric measurements (body weight, height, waist circumference, systolic blood pressure, diastolic blood pressure). Fasting laboratory analysis performed at the time of admission to the hospital that were assessed included: complete blood count using an automated hematology analyzer (Sysmex XN-550; Sysmex Corporation, Kobe, Japan) and biochemical tests. The latter parameters, which included fasting glucose levels, total cholesterol, HDL-C, and TG levels, were measured using an automatic analyzer (Hitachi 747; Hitachi, Tokyo, Japan). Low-density lipoprotein cholesterol (LDL-C) plasmatic levels were calculated according to the Friedewald equation [106]. Insulinemia was analyzed by means of automated chemiluminescent assay (Cobas E 411-Roche; Tokyo, Japan). Given these retrospectively available laboratory data, the following CBC-derived indices were calculated: neutrophil count/lymphocyte count (NLR), platelet count/lymphocyte count (PLR), platelet count * NLR (SII), and neutrophil count × monocyte/lymphocyte count (SIRI); HOMA-IR was calculated using the following formula: fasting insulin [mIU/L] × fasting glucose [mg/dL]/405 [107]. The triglyceride-to-high-density lipoprotein cholesterol (TG:HDL-C) ratio was calculated as the amount of triglycerides (mg/dL) divided by the HDL-C level (mg/dL) [108], and non-high-density lipoprotein cholesterol (non-HDL-C), by subtracting the HDL-C (mg/dL) from the total cholesterol (mg/dL) [109].

### 4.3. Statistical Analysis

The two study groups were defined using descriptive statistics (percentage, mean, median, standard deviation (SD), interquartile range). Visual (histograms, probability plots) and analytical methods (Shapiro–Wilk test) were used to assess the extent to which the data followed a normal distribution. Numerical variables with a normal distribution were expressed as the mean ± SD, and an independent Student’s *t*-test was used to analyze the differences between the two groups. Numerical variables without normal distribution were plotted as medians (25th and 75th interquartile range) and the Mann–Whitney *U* test was used for comparison of the two groups. Categorical variables were presented as the number (percentage), and Chi-squared testing was used to compare such variables. Spearman’s rank correlation coefficient (*r*) was used to assess the relationships between the SII and other variables. Univariate logistic regression analysis was used to determine the association of laboratory markers of inflammation (WBC, NLR, PLR, SII, SIRI) with the presence of metabolic syndrome. Multiple linear regression analysis was performed to explore the relationship between the SII and individual components of metabolic syndrome. Finally, the diagnostic value of the SII in identifying metabolic syndrome patients was determined by a receiver operating characteristic (ROC) curve. Youden’s index (calculated as sensitivity + specificity − 1) determined proper cut-off values for several biomarkers. ROC curve comparisons were plotted for the SII, NLR, PLR, SIRI, HOMA-IR, TG/HDL-C ratio, and non-HDL-C to compare the discrimination ability of those variables in identifying patients with metabolic syndrome. To compare the results, the area under the curve (AUC) in the ROC analysis was determined (values between 0.5 and 1.0 were considered significant and values closer to 1.0 indicated the most significant relationship). All statistical analyses were performed using Statistical Package for Social Sciences software (SPSS v28.0.0.0.; IBM Corp: Armonk, NY, USA). A *p*-value (two-tailed) < 0.05 was considered statistically significant.

## 5. Conclusions

The present study interpreted complementary laboratory data regarding children with metabolic syndrome. To the best of our knowledge, it is the first to examine the relationship between the SII and cardiometabolic risk biomarkers in obese children with metabolic syndrome, highlighting its role as an additional measure of cardiometabolic instability and confirmatory proof of the timely initiation of the inflammatory process in obese children with MetS. 

These findings have clinical relevance, especially in pre-hospital settings, because they may improve the routine preventative care and diagnosis of metabolic syndrome in children with obesity. Therefore, this index, which is inexpensive and universally available in primary care settings, could represent an attractive alternative or addition to frequently assessed inflammatory biomarkers.

## Figures and Tables

**Table 1:** Clinical and biochemical characteristics of the patients.

Parameter	Non-MetS (*n* = 125)	MetS (*n* = 66)	*p*-Value
Age (years)	12 (11–15)	13 (11–15)	0.104 ^U^
% Males (*n*)	58.4 (73)	57.6 (38)	0.913 ^χ2^
BMI (kg/m^2^)	29.9 (27.9, 32.2)	32.9 (29.3, 37.9)	**<0.001** **^U^**
WC (cm)	93.7 ± 13.7	104.1 ± 18.9	**<0.001** **^t^**
SBP (mmHg)	115 (103, 123)	125 (111, 140)	**<0.001** **^U^**
DBP (mmHg)	69.8 ± 11	78.3 ± 12.9	**<0.001** **^t^**
WBCs (×10^3^/mm^3^)	7.23 (6.07, 8.38)	9.42 (7.90, 10.46)	**<0.001** **^t^**
Neutrophils (×10^3^/mm^3^)	3.56 (2.90, 4.53)	5.47 (4.18, 6.65)	**<0.001** **^t^**
Lymphocytes (×10^3^/mm^3^)	2.56 ± 0.83	2.66 ± 0.81	0.789 ^t^
Thrombocytes (×10^3^/mm^3^)	296 ± 63.94	340.1 ± 83.3	**<0.001** **^t^**
Monocytes (×10^3^/mm^3^)	0.64 (0.53–0.79)	0.71 (0.58–0.82)	0.103 ^U^
Glycemia (mg/dL)	89.82 (84.87–94.05)	91.89 (86.22–100.93)	**0.034** **^U^**
Insulinemia (uIU/mL)	16.43 (12.83–25.37)	25.36 (15.61–42.72)	**<0.001** **^U^**
TG (mg/dL)	82.37 (56.90–106.28)	152.78 (102.29–185.33)	**<0.001** **^U^**
Cholesterol (mg/dL)	154.33 ± 33.27	162.21 ± 38	0.148 ^t^
HDL-C (mg/dL)	46.65 ± 10.30	36.47 ± 8.64	**<0.001** **^t^**
LDL-C (mg/dL)	88.28 ± 31.38	93.37 ± 33.03	0.431 ^t^
HOMA-IR	3.52 (2.68–5.89)	6.40 (3.66–9.10)	**<0.001** **^U^**
TG:HDL-C	1.77 (1.05–2.74)	4.13 (2.71–5.55)	**<0.001** **^U^**
Non-HDL-C	106 (78.5–125.3)	121.4 (99.1–144.4)	**<0.001** **^U^**

^U^ Mann–Whitney test, ^χ2^ chi-squared, and ^t^ independent sample *t*-test. Data are expressed as the mean ± standard deviation, median (interquartile range, IQR), or percentage (*n*, %). Abbreviations: BMI, body mass index; WC, waist circumference; SBP, systolic blood pressure; DPB, diastolic blood pressure; WBC, white blood cell count; TG, triglyceride; HDL-C, high-density lipoprotein cholesterol; LDL-C, low-density lipoprotein cholesterol; HOMA-IR, Homeostasis Model Assessment of Insulin Resistance; TG:HDL-C, triglyceride-to-high-density lipoprotein cholesterol; non-HDL-C, non-high-density lipoprotein cholesterol. Statistically significant differences, with a probability value of *p* < 0.05, are represented in bold.

**Table 2:** Inflammatory parameters of the study groups.

Parameter	Non-MetS (*n* = 123)	MetS (*n* = 68)	*p*-Value
NLR	1.36 (1.12–1.77)	1.90 (1.58–2.67)	**<0.001** ^t^
PLR	117.24 (93.75–140.20)	128.25 (108.10–169.02)	**<0.001** ^t^
SII	363.4 (277.9–477.01)	715.87 (472.98–909.15)	**<0.001** ^t^
SIRI	0.890 (0.633–1.312)	1.380 (0.929–2.030)	**<0.001** ^t^

^t^ Independent sample *t*-test. Data are expressed as the median (interquartile range, IQR). Abbreviations: NLR, neutrophil-to-lymphocyte ratio; PLR, platelet-to-lymphocyte ratio; SII, systemic immune-inflammation index; SIRI, systemic inflammation response index. Statistically significant differences, with a probability value of *p* < 0.05, are represented in bold.

**Table 3:** Correlations of SII with markers of cardiometabolic risk in obese children.

	HOMA-IR			TG:HDL-C			Non-HDL-C		
Item	r_S_	*p*	95% CI	r_S_	*p*	95% CI	r_S_	*p*	95% CI
SII	0.274	<0.001	0.115–0.419	0.239	0.002	0.083–0.383	0.18	0.021	0.024–0.334

**Table 4:** Binomial regression analysis regarding independent predictors of MetS in obese children.

Bivariate Analysis		
Variable	OR (95% CI)	*p*-Value
SII	1.005 (1.003–1.008)	**<0.001**
SIRI	1.071 (0.938–1.224)	0.311
HOMA-IR	0.962 (0.731–1.267)	0.783
Non-HDL-C	0.993 (0.976–1.010)	0.423
TG:HDL-C	2.884 (1.825–4.555)	**<0.001**

Abbreviations: SII, systemic immune-inflammation index; SIRI, systemic inflammation response index; HOMA-IR, Homeostasis Model Assessment of Insulin Resistance; non-HDL-C, non-high-density lipoprotein-cholesterol; TG:HDL-C, triglyceride-to-high-density lipoprotein cholesterol. Statistically significant differences, with a probability value of *p* < 0.05, are represented in bold.

**Table 5:** Relationship between independent MetS components and the SII in obese children.

Variable	ß (95% CI)	*p*-Value
WC (cm)	1.578 (0.281–6.552)	**0.033**
SBP (mmHg)	2.076 (−3.337–4.9130)	0.705
DBP (mmHg)	2.789 (−2.931–8.155)	0.352
Glycemia (mg/dL)	1.593 (−5.674–0.656)	0.119
HDL-C (mg/dL)	2.398 (−8.355–1.176)	0.138
TG (mg/dL)	0.399 (−0.799–0.786)	0.987

Note. Model = “Enter” method in SPSS statistics; ß = standardized coefficient; CI = confidence interval. Abbreviations: WC, waist circumference; SBP, systolic blood pressure; DPB, diastolic blood pressure; HDL-C, high-density lipoprotein cholesterol; TG, triglyceride. Statistically significant differences, with a probability value of *p* < 0.05, are represented in bold.

**Table 6:** Comparison of inflammatory and cardiometabolic indices in discriminating MetS.

	AUC	SE	95% CI	Sensitivity	Specificity	Cut-Off	*p*-Value
SII	0.843	0.031	0.782–0.904	0.83	0.63	426.8	**<0.001**
SIRI	0.708	0.039	0.630–0.785	0.69	0.67	1.123	**<0.001**
HOMA-IR	0.689	0.046	0.600–0.778	0.66	0.62	4.24	**<0.001**
Non-HDL-C	0.652	0.044	0.565–0.739	0.63	0.61	107.7	**0.001**
TG:HDL-C	0.816	0.037	0.744–0.888	0.81	0.50	1.786	**<0.001**

Abbreviations: SII, systemic immune-inflammation index; SIRI, systemic inflammation response index; HOMA-IR, Homeostasis Model Assessment of Insulin Resistance; non-HDL-C, non-high-density lipoprotein-cholesterol; TG:HDL-C, triglyceride-to-high-density lipoprotein cholesterol; AUC, area under the curve; SE, standard error; 95% CI, 95% confidence interval. Statistically significant differences, with a probability value of *p* < 0.05, are represented in bold.

## Data Availability

The data are not publicly available due to reasons of privacy.

## Supplementary Materials

The following supporting information can be downloaded at: https://www.mdpi.com/article/10.3390/ijms24098414/s1.
