# Supplementary material for: Assessing the Relationship between Systemic Immune-Inflammation Index and Metabolic Syndrome in Children with Obesity
Source: Int J Mol Sci. 2023 May 8;24(9):8414. doi: 10.3390/ijms24098414 (PMC10179406; doi:10.3390/ijms24098414)
Supplement: Supplementary file 1 [file ijms-24-08414-s001.zip › ijms-2350140-supplementary.pdf]

Table S1: Correlations of inflammatory indices with cardiometabolic risk indices in obese children

	HOMA-IR			TG:HDL-C			non-HDL-C		
Items	r_S	p	C.I.95%	r_S	p	C.I.95%	r_S	p	C.I.95%
**NLR**	0.225	0.005	0.063-0.376	0.150	0.059	-0.010-0.302	0.121	0.131	-0.041-0.276

	HOMA-IR			TG:HDL-C			non-HDL-C		
Items	r_S	p	C.I.95%	r_S	p	C.I.95%	r_S	p	C.I.95%
**PLR**	-0.015	0.918	-0.156-0.173	-0.015	0.852	-0.174-0.145	-0.006	0.939	-0.167-0.155

	HOMA-IR			TG:HDL-C			non-HDL-C		
Items	r_S	p	C.I.95%	r_S	p	C.I.95%	r_S	p	C.I.95%
**SIRI**	0.251	0.002	0.091-0.400	0.162	0.042	0.001-0.315	0.144	0.073	-0.018-0.300
